# Structural determinants of depressive symptoms among refugees and host communities in South Sudan: evidence from explainable machine learning

**DOI:** 10.3389/fpubh.2026.1778986

**Published:** 2026-04-14

**Authors:** Hyojin Im, Ashwag Abdulrahim

**Affiliations:** 1 School of Social Work, Virginia Commonwealth University, Richmond, VA, United States; 2 Department of Health Administration, College of Health Professions, Virginia Commonwealth University, Richmond, VA, United States

**Keywords:** depressive symptoms, explainable machine learning, forced displacement, humanitarian settings, refugees and host communities, SHAP (SHapley Additive Explanations), South Sudan, structural determinants of mental health

## Abstract

**Background:**

Depressive symptoms in displacement settings are often framed as consequences of refugee status, which can obscure the shared structural conditions emphasized in the Social Determinants of Health (SDoH) framework that shape mental health for refugees and host communities and limit the identification of practical intervention targets. This study examines the relative contribution of health, socioeconomic, protection, and contextual factors to depressive symptom severity among adults living in displacement-affected settings in South Sudan.

**Methods:**

We analyzed nationally representative data from 3,055 adults (2,066 refugees, mean displacement duration 11.15 years; 989 host community members) from the 2023 Forced Displacement Survey. Depressive symptom severity was measured using the PHQ-9. We compared Elastic Net regression, Random Forests, and Extreme Gradient Boosting (XGBoost) using 10 × 5 nested cross-validation. The best-performing model was interpreted using SHapley Additive exPlanations (SHAP) to estimate the marginal contribution of each predictor in PHQ units.

**Results:**

Mean depressive symptom severity was low to moderate overall (*M* = 4.43, SD = 5.00) and did not differ by population type (*p* = 0.783). XGBoost showed the highest predictive performance [Root Mean Squared Error (RMSE) = 4.47, *R*^2^ = 0.247], significantly outperforming Elastic Net regression (*p* = 0.006). Model explanations identified self-rated health status as the dominant predictor (19.3% of total importance), followed by perceived community violence (11.1%), perceived poverty (9.6%). Age (9.3%), discrimination (9.2%), food insecurity (8.6%), and citizenship (8.2%, pooled model only) contributed at moderate levels, whereas social protection (3.4%) and remittances (0.7%) contributed minimally. Predictor profiles were broadly similar across refugee and host models, with differences primarily in magnitude rather than rank ordering.

**Conclusion:**

Depressive symptoms in South Sudan appear to be structured primarily by health, material hardship, and protection-related gradients rather than refugee status *per se*. Findings support integrated, area-based public health responses that link mental health support with primary health care access, poverty-oriented assistance, and protection and safety interventions rather than programming organized primarily around legal status distinctions.

## 1 Introduction

South Sudan has emerged as a critical refugee host since its 2011 independence, shaped by persistent instability and climate shocks in both the country and the neighboring region. By April 2024, the country sheltered over 450,000 refugees, primarily from Sudan, the Democratic Republic of Congo, and Ethiopia (1), with numbers surging following Sudan's April 2023 conflict escalation. This displacement is not an isolated event but part of a much longer history of political conflict rooted in the First and Second Sudanese Civil Wars (1955–2005), driven by religious-ethnic divisions, economic marginalization, and the imposition of Sharia law (2). Even after independence in 2011, underlying tensions persisted, culminating in the December 2013 South Sudanese Civil War that destabilized the new nation (2). The April 2023 outbreak of conflict between the Sudanese Armed Forces (SAF) and the Rapid Support Forces (RSF) illustrates the persistence of instability, producing the world's largest displacement crisis, with an estimated 150,000 deaths by June 2024 and 12 million people displaced by July 2025 (3). Nearly one million of these have sought refuge in South Sudan, placing immense pressure on already fragile resources and humanitarian systems (1, 4).

South Sudan's humanitarian emergency is sustained by mutually reinforcing violence against civilians and environmental stressors that steadily degrade livelihoods, governance, and health systems. Indiscriminate attacks, arbitrary detention, sexual violence, and extrajudicial killings have been widely documented during the Sudan war that spills across borders and drives new arrivals into South Sudan, with rights groups and international observers warning of ethnic cleansing and grave crimes (5, 6). These pressures intersect with climate hazards that are among the world's most acute. South Sudan ranks at the extreme end of climate vulnerability, and the livelihoods of the vast majority, commonly estimated at 85–95 percent, depend on rain-fed subsistence farming and pastoralism that are highly sensitive to hydrometeorological shocks (7–9). In recent years, multi-year floods have repeatedly affected on the order of 750,000 to more than one million people annually, while drought episodes in the southeast have devastated crops and herds; these shocks intensify competition over grazing land and water, feed cattle raiding and inter-communal violence, and trigger secondary displacement that further burdens host communities (8). The result is a cumulative displacement and insecurity dynamic in which environmental hazards are not background conditions but key drivers that erode community resilience and undermine fragile peace processes (10).

These vulnerabilities are further compounded by a precipitous economic decline. While real incomes were decimated during the civil war, enduring macroeconomic instability continues to erode household resilience. Since early 2024, food prices rose approximately 155 percent and the official exchange rate depreciated by about 160 percent, placing basic goods out of reach for much of the population and increasing dependence on humanitarian assistance in a country that is heavily import-reliant (11, 12). Health systems are simultaneously weakened by chronic underinvestment and direct violence against care. South Sudan continues to report one of the world's highest maternal mortality ratios, reflecting severely limited access to skilled birth attendance and emergency obstetric care; recurrent outbreaks of cholera, measles, and malaria add substantial burden (13). Attacks on health facilities in Upper Nile and Unity states have forced closures and looting of medicines, abruptly cutting services for an estimated 150,000 people and demonstrating how insecurity directly removes lifesaving capacity from already thin service networks (14, 15).

Within this structural context, mental health needs are large, persistent, and closely tied to cumulative trauma, displacement, and deprivation. Early post-conflict work in Juba found substantial depressive symptomatology alongside extensive trauma exposure and material deprivation, with clear associations between cumulative events and worse mental health (16). Population-based surveys in Greater Bahr el Ghazal linked multiple traumatic events and current hardship to probable PTSD and depression, including comorbidity that signals severe functional impact (17, 18). At the same time, community studies emphasize locally salient idioms of distress such as “overthinking,” and show that gendered violence, displacement histories, and livelihood loss shape symptom patterns, especially among women and adolescents (19, 20). Instrument development tailored to South Sudan further underscores the centrality of context by validating a 24-item scale that captures common idioms and performs well psychometrically, which strengthens inference when studying non-clinical populations (21). Recent systematic reviews and meta-analyses estimate that approximately one in four adults in conflict-affected settings experiences depression or PTSD at any time, and that prevalence among refugees in East Africa is high yet highly heterogeneous, partly reflecting measurement tools, exposure profiles, and living conditions (22–24). UNHCR's cultural formulation review confirms that mental health is inseparable from security, food access, and social support, with stigma and limited services restricting help-seeking among South Sudanese displaced populations (25).

This prevailing burden within South Sudan is significantly amplified by the trauma profiles of incoming refugees, whose mental health reflects both the chronic instability and acute violence of the Sudan conflict. Even prior to the April 2023 escalation, studies in Sudan had already indicated elevated morbidity. Depression affected 35.6% patients with chronic conditions (26) while 24.3% of long-term internally displaced persons (IDPs) met criteria for major depressive disorder and 23.6% for generalized anxiety disorder (27). Following the onset of the 2023 conflict, evidence shows a sharp rise in psychological distress across Sudan. A nationwide study found that more than half of adults reported moderate to very severe depression or anxiety symptoms, with higher levels among females and those in war-affected regions (28). Among adolescents, rates of major depressive disorder surged to 67.7% (29). In Khartoum, 70% of civilians exhibited symptoms of depression and 57.3% reported anxiety symptoms (30). Similarly, among IDPs in White Nile State, the prevalence of depression was 18%, anxiety 14%, and PTSD 20%. Among refugees and displaced populations directly exposed to conflict, 36.6% reported PTSD, with refugees experiencing higher levels of trauma and depressive symptoms compared to IDPs and non-displaced individuals (31).

Across the diverse populations studied in Sudan, several factors were consistently associated with higher risk of depression and anxiety. These include female gender (31–34), displacement and longer duration of displacement (27–29, 31), exposure to traumatic events (29), and lower socioeconomic status (26, 31, 32). Physical comorbidities also contributed to mental health vulnerability, with studies identifying anemia among adolescents (35) and coexisting chronic diseases among patients with diabetes (26) as significant risk factors for anxiety and depression, respectively.

Despite increasing evidence, critical gaps impede the development of targeted, evidence-based interventions in the complex hosting environment of South Sudan. First, much of the empirical work in South Sudan and the region has examined depressive symptoms in relation to a limited subset of determinants at a time, often focusing on trauma exposure or current stressors, which makes it difficult to compare the relative influence of health, socioeconomic status, perceived safety and violence, social protection, and place characteristics when considered jointly (16, 22, 36). Second, refugee samples remain more common than inclusive designs that also enroll host community members who share many exposures, including food insecurity and local violence, while holding different legal and social positions; recent guidance and syntheses call for more host–refugee comparative designs to inform area-based responses (25, 37). This need for host–refugee comparative designs aligns with a broader Social Determinants of Mental Health (SDoMH) literature demonstrating that common mental disorders are structured primarily by social and material gradients rather than categorical group membership alone. The WHO Commission on Social Determinants of Health framework distinguishes structural determinants that shape opportunity and resource distribution from intermediary determinants that translate those structures into lived exposure and daily stress (38). Recent population-level analyses similarly show that area-level deprivation and health system access account for substantial variation in depressive symptom burden, often exceeding differences attributable to migration or legal status categories (39, 40). Third, comparatively fewer studies apply predictive and explainable modeling to quantify how plausible changes in modifiable factors map onto depressive symptoms in outcome units, a gap noted in broader mental health methods reviews and only sparsely addressed in humanitarian settings despite the availability of transparent machine learning approaches (41–43). Addressing these gaps requires designs that model multi-domain determinants together, explicitly compare patterns across refugee and host groups, and translate complex models into interpretable quantities that can guide integrated protection, primary care, and livelihood interventions.

### 1.1 Conceptual framework

This study is grounded in the WHO Commission on Social Determinants of Health (SDoH) framework, which distinguishes between structural determinants that shape the distribution of power, resources, and opportunity and intermediary determinants that translate these structural conditions into material, psychosocial, and behavioral exposures (38, 44). In the South Sudan context, structural determinants refer to upstream positioning factors such as legal status and rurality that condition access to services, protection, mobility, and livelihood opportunities within a fragile governance and humanitarian system. These positions shape the opportunity landscape of daily life. Intermediary determinants include material hardship (e.g., perceived poverty, food insecurity), community-level insecurity (e.g., sense of safety, perceived community violence), and proximal individual health conditions such as self-rated health, which may function as a mediator or proxy for functional limitation rather than a purely exogenous determinant. These factors represent the lived manifestations of structural positioning and operate as pathways linking structural context to psychological outcomes (45). We conceptualize depressive symptoms as embedded within the structural and material conditions that shape everyday functioning in displacement affected settings. Because these processes are mutually reinforcing, particularly for proximal indicators such as self-rated health, we do not frame the analysis as causal isolation. Instead, we estimate the relative salience of structural positioning and intermediary exposures in differentiating symptom severity within a uniformly high adversity environment, treating self-rated health as an indicator of functional limitation and embodied hardship that may be reciprocally related to depressive symptoms.

## 2 Methods

### 2.1 Dataset

This study draws on the *Forced Displacement Survey (FDS)—South Sudan 2023*, a nationally representative household survey conducted jointly by UNHCR, the World Bank, and South Sudan's National Bureau of Statistics. The FDS was designed to capture the living conditions, socioeconomic status, and wellbeing of forcibly displaced populations, including refugees, returnees, and host communities, alongside non-displaced comparison groups. The FDS employed a multi-stage, stratified sampling design. In the North, sampling used the Google Open Buildings database with stratified systematic selection of building units; in the South, sampling used UNHCR's proGres registration database with stratified systematic selection of registration cases. Within each sampled household or registration case, one adult was randomly selected for individual interview. Because the analytic dataset included a single adult per sampled unit, no intra-household clustering was present in the outcome. Survey weights were constructed to produce nationally representative estimates of registered refugees and host communities. In total, the survey included more than 30,000 individuals across ~6,000 households, encompassing displaced (refugees, IDPs, and returnees) and host community members. For the present analysis, the analytic subsample comprised 3,055 adult participants with observed PHQ-9 scores and covariates after addressing low levels of missingness via multiple imputation.

**Figure 1:**
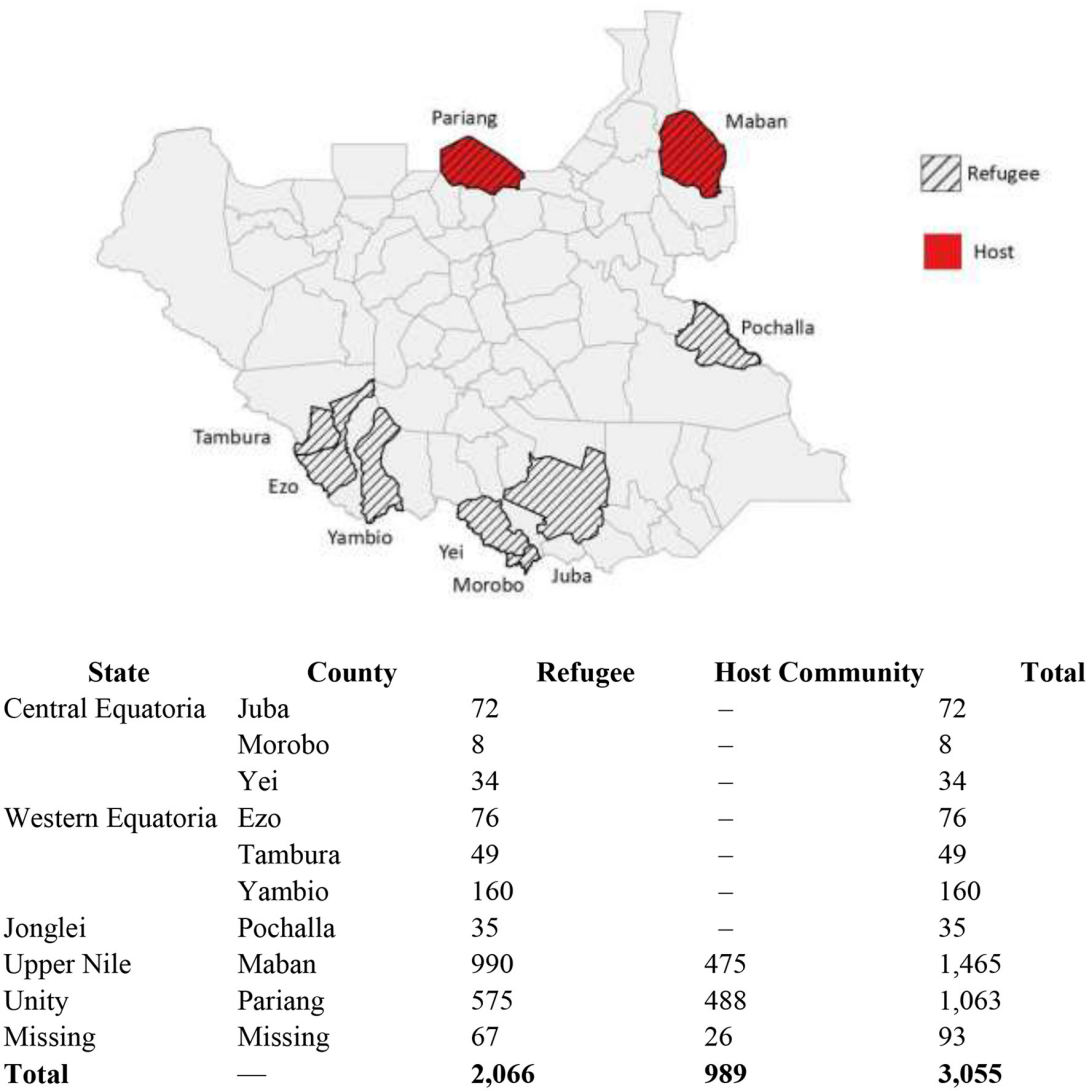
Geographic distribution of study participants.

Figure 1 shows geographic distribution of the final study sample used in our analysis. Participants were distributed across nine counties. Refugee individuals were selected from Pariang, Maban, Juba, Morobo, Yei, Pochalla, Ezo, Tambura, and Yambio, while participants from the host community lived in proximity to refugees in Pariang and Maban. Further details on the study design, sampling strategy, and participant recruitment procedures are provided in the *Forced Displacement Survey—South Sudan 2023* technical documentation (46).

### 2.2 Measures

#### 2.2.1 Dependent variable: depressive symptoms

Depressive symptom severity was assessed using the Patient Health Questionnaire (PHQ-9), a widely used and validated self-report screening instrument that has been applied extensively in humanitarian and low-resource settings. The PHQ-9 assesses the frequency of nine depressive symptoms experienced over the preceding 2 weeks, including affective, cognitive, and somatic domains (e.g., “Feeling down, depressed, or hopeless”). Response options range from 0 (“not at all”) to 3 (“nearly every day”), yielding a total score ranging from 0 to 27, with higher scores indicating greater symptom severity. PHQ scores were treated as a continuous outcome variable in all analyses.

#### 2.2.2 Health and socioeconomic determinants

Self-rated health status was measured using a single-item global health question: “How is your health in general?” Responses were recorded on a 5-point ordinal scale ranging from 1 (“very bad”) to 5 (“very good”), with higher values indicating better perceived health. Perceived poverty level captured subjective economic position by asking respondents to compare their household's current situation to a minimum income threshold. Responses were recorded on a 5-point ordinal scale ranging from 1 (“a lot above minimum income”) to 5 (“a lot below minimum income”), such that higher scores reflected greater perceived poverty. Food insecurity was measured using the nine-item Household Food Insecurity Access Scale (HFIAS), which assesses the severity of household food insecurity (e.g., anxiety over food supply to severe hunger experiences). Coded binary, with higher HFIAS scores reflecting more severe food insecurity.

#### 2.2.3 Community-level exposures

Sense of safety was measured using a single item assessing perceived personal safety: “How safe do you feel walking alone in your area after dark?” Responses were recorded on a 4-point ordinal scale ranging from 1 (“very safe”) to 4 (“very unsafe”), with higher scores indicating lower perceived safety. Perceived community violence was operationalized as a composite score derived from five items assessing the frequency of neighborhood level violent events, with higher scores reflecting greater perceived exposure. Internal consistency was acceptable (Cronbach's α = 0.71). Perceived discrimination was measured using nine items assessing experiences of harassment or unfair treatment (e.g., being treated rudely or called names), with total scores ranging from 0 to 37 (Cronbach's α = 0.90).

#### 2.2.4 Legal, assistance, and contextual factors

Legal status distinguished respondents who were South Sudanese nationals from those who were nationals of other countries, primarily Sudan, the Democratic Republic of Congo, and Ethiopia. This variable was included to capture differences in legal positioning within the host country.

Social protection was measured as a binary indicator (yes/no) denoting whether the respondent or their household received formal assistance, including cash transfers, food aid, or other in-kind benefits. Receiving remittances was also coded as a binary variable (yes/no), indicating whether the household received financial support from relatives or others living abroad.

#### 2.2.5 Sociodemographic covariates

Age was measured as a continuous variable in years. Sex was recorded as a binary variable (male or female). Rurality classified respondents' place of residence into urban, peri-urban, or rural categories.

Residence type distinguished whether respondents lived in a camp, settlement, or neither.

### 2.3 Data analysis

#### 2.3.1 Descriptive analysis

Descriptive analyses were conducted to summarize the characteristics of the study population overall and by population type (refugees and host community members). Continuous variables were summarized using survey-weighted means, and categorical variables using survey-weighted percentages. Group differences were assessed using survey-adjusted *t*-tests for continuous variables and survey-adjusted χ^2^ tests for categorical variables. Missingness was low across analytic covariates (all < 5%; See Supplementary Table S1). We used multivariate imputation by chained equations (MICE) to impute missing predictor values. To prevent information leakage, imputation and all preprocessing steps, including encoding, were performed within each outer-fold training set under the 10 × 5 nested cross-validation framework, and the learned transformations were then applied to the corresponding held-out fold.

#### 2.3.2 Predictive modeling and model interpretation

All analyses were conducted in a supervised machine learning framework designed to predict depressive symptom severity (PHQ-9), from a set of sociodemographic, health, and contextual predictors. We compared three algorithms with distinct strengths. The first was Extreme Gradient Boosting (XGBoost), a tree-ensemble method that sequentially builds decision trees to optimize residual errors and has demonstrated high predictive performance in diverse health and social science applications (47). The second was Random Forests, an ensemble of decision trees generated through bootstrap aggregation, known for its robustness to noise and multicollinearity (48). The third was Elastic Net regression, a penalized linear approach that combines LASSO and ridge penalties to handle correlated predictors and perform variable selection (49). To avoid optimistic bias in model evaluation, we used a nested cross-validation (CV) procedure (50, 51). Specifically, we implemented a 10 × 5 nested CV design, in which each of the 10 outer folds was held out in turn for model testing, while hyperparameter tuning was performed within an inner five-fold CV. This design ensured that parameter optimization and performance estimation were separated, thereby producing robust estimates of generalization error in a sample of moderate size. Model performance was evaluated using three standard metrics. Root Mean Squared Error (RMSE) quantified predictive accuracy while penalizing larger deviations more heavily. Mean Absolute Error (MAE) captured the typical magnitude of prediction error. The proportion of variance explained (*R*^2^) was used to summarize overall model fit. To statistically compare model performance, we applied paired Wilcoxon signed-rank tests to the outer-fold RMSE distributions, a non-parametric approach appropriate for repeated cross-validation results (52).

Descriptive analyses were survey-weighted to account for stratification and unequal selection probabilities. Predictive machine learning models, however, were trained without sampling weights because the estimand of interest was the conditional expectation of depressive symptom severity given observed covariates within the analytic sample, consistent with a predictive rather than design-based inferential objective (48, 53). In tree-ensemble methods, incorporating highly variable survey weights can increase variance and reduce model stability by disproportionately emphasizing high-weight observations (54). Accordingly, models were trained without sampling weights to align with a predictive estimand and to avoid the disproportionate influence of high-weight observations, which can increase variance and reduce model stability in gradient-boosted ensembles. As a sensitivity check, model performance was compared under stratified fold construction by county and population type, yielding substantively similar predictive accuracy estimates. In addition to estimating models in the pooled sample, we fit parallel models separately for refugees and host community members using the identical nested cross validation procedure and hyperparameter tuning strategy. This allowed assessment of whether predictor importance and model performance differed meaningfully across population groups, while maintaining a consistent modeling framework.

Beyond predictive accuracy, we also applied SHapley Additive exPlanations (SHAP), a game-theoretic approach that decomposes each model prediction into additive contributions of individual features, thereby enhancing interpretability (41). SHAP values are expressed in outcome units (PHQ points), permitting direct interpretation of how much each predictor increased or decreased the predicted depression score for a given participant relative to the model baseline. Global feature importance was assessed by averaging the absolute SHAP values across all participants, which provided a ranking of predictors by their overall influence on model output. SHAP values were computed for both pooled and group specific models, allowing comparison of predictor rankings and contribution patterns across refugees and host community members. We also examined heterogeneity in predictor effects by visualizing the distribution of SHAP values, which illustrated whether a predictor consistently moved predictions in one direction or whether its effects varied across subgroups of participants. Two complementary SHAP visualizations were used to summarize feature contributions at both the individual and aggregate levels. First, the distribution of SHAP values was examined across all predictors and participants to characterize the direction and magnitude of each predictor's contribution to PHQ predictions. Second, a SHAP beeswarm summary was used to rank predictors by mean absolute SHAP value while displaying the dispersion of contributions across individuals, thereby capturing global importance alongside heterogeneity in effects. Together, these procedures supported identification of the most influential predictors of depressive symptoms while enhancing model interpretability and transparency, consistent with recommended practices in explainable machine learning (43, 55).

#### 2.3.3 Sensitivity analysis for measurement overlap

To assess potential measurement overlap between self-rated health and depressive symptom severity, we conducted sensitivity analyses using alternative outcome specifications. An affective-cognitive PHQ-7 score was constructed by subtracting the two somatic items related to sleep disturbance and fatigue from the PHQ-9 total. Models were re-estimated both with and without self-rated health to quantify its incremental predictive contribution. A two-item somatic subscore was also examined to assess expected overlap effects. All analyses were conducted using the same nested cross-validation and SHAP-based interpretability framework as the primary models.

## 3 Results

### 3.1 Sample profile and key differences between refugees and host community members

The analytic sample comprised 3,055 participants, including 2,066 (67.6%) refugees and 989 (32.4%) host community members (Table 1). Depressive symptom severity was low to moderate overall (PHQ-9 mean 4.43, SD 5.00) and did not differ by population type (refugees mean 4.39, SD 5.46; host community mean 4.49, SD 4.13; *p* = 0.783). Mean age was similarly comparable (32.51 years, SD 12.98; *p* = 0.643). Among refugees, time in South Sudan since displacement averaged 11.15 years (SD 2.83), indicating a predominantly protracted displacement context rather than a newly arrived population.

**Table 1:** Sample characteristics by population type.

Variable	Population			
	Refugees	Host community	Total	Test
*N* (%)	2,066 (67.6%)	989 (32.4%)	3,055 (100.0%)	
PHQ-9 (mean, SD)	4.39 (5.46)	4.49 (4.13)	4.43 (5.00)	0.783
Years in SSD since displacement^*^ (mean, SD)	11.15 (2.83)	NA	11.15 (2.83)	
Age (mean, SD)	32.48 (13.34)	32.56 (12.22)	32.51 (12.98)	0.643
Health status (*N*, %)				
Very bad	53 (2.70%)	134 (11.82%)	187 (6.07%)	<0.001
Bad	200 (10.16%)	186 (20.01%)	386 (13.80%)	
Neither good or bad	294 (14.59%)	194 (17.66%)	488 (15.72%)	
Good	985 (48.49%)	365 (38.40%)	1,350 (44.77%)	
Very good	533 (25.05%)	110 (12.10%)	643 (19.64%)	
Sex (*N*, %)				
Female	1,061 (55.8%)	535 (51.1%)	1,596 (56.6%)	<0.001
Male	840 (44.2%)	578 (51.91%)	1,418 (47.05%)	
Marital status (*N*, %)				
Married	1,240 (60.01%)	701 (66.2%)	1,941 (63.8%)	0.16
Non-formal union	14 (0.46%)	2 (0.24%)	16 (0.38%)	
Divorced	156 (4.87%)	27 (2.7%)	183 (4.05%)	
Widow	106 (3.63%)	36 (3.45%)	142 (3.56%)	
Never married	540 (31.03%)	219 (27.45%)	759 (29.71%)	
Rurality (*N*, %)				
Rural	1,338 (63.27%)	718 (69.97%)	2,056 (65.74%)	<0.001
Peri-urban	490 (23.05%)	249 (27.46%)	739 (24.68%)	
Urban	237 (13.68%)	21 (2.57%)	258 (9.58%)	
Residence (*N*, %)				
Camp	1,844 (89.29%)	6 (0.76%)	1,850 (56.69%)	<0.001
Settlement	200 (9.81%)	854 (86.5%)	1,054 (38.05%)	
Neither camp nor settlement	20 (0.90%)	127 (12.74%)	147 (5.26%)	
Citizenship (*N*, %)				
South Sudan	77 (4.29%)	963 (98.69%)	1,040 (39.32%)	<0.001
Sudan	1,544 (80.03%)	4 (0.73%)	1,548 (50.60%)	
Democratic Republic of the Congo	242 (9.61%)	0 (0.0%)	242 (6.05%)	
Central African Republic	56 (2.57%)	0 (0.0%)	56 (1.62%)	
Ethiopia	83 (3.39%)	0 (0.0%)	83 (2.13%)	
Others	5 (0.11%)	4 (0.58%)	9 (0.29%)	
Received social protection (*N*, %)				
Yes	1,195 (53.15%)	109 (10.64%)	1,304 (37.48%)	<0.001
No	866 (46.85%)	876 (89.36%)	1,742 (62.52%)	
Poverty level (*N*, %)				
A lot below	1,076 (59.16%)	574 (61.73%)	1,650 (60.05%)	0.158
A little below	540 (23.81%)	227 (25.49%)	767 (24.39%)	
About the same	279 (12.21%)	63 (10.00%)	342 (11.45%)	
A little above	75 (3.45%)	14 (1.50%)	89 (2.78%)	
A lot above	21 (1.37%)	11 (1.27%)	32 (1.33%)	
Perceived poverty level (*N*, %)				
Below minimum	1,616 (82.97%)	801 (87.23%)	2,417 (84.44%)	0.062
At/above minimum	375 (17.03%)	88 (12.77%)	463 (15.56%)	
Sense of safety (*N*, %)				
Very safe	1,086 (52.50%)	466 (53.86%)	1,552 (53.00%)	<0.001
Safe	452 (21.63%)	168 (14.83%)	620 (19.09%)	
Unsafe	177 (11.69%)	221 (19.89%)	398 (14.74%)	
Very unsafe	261 (14.19%)	108 (11.43%)	369 (13.16%)	
Sense of safe—binary (*N*, %)				
Not safe	438 (25.88%)	329 (31.32%)	767 (27.90%)	0.029
Safe	1,538 (74.12%)	634 (68.68%)	2,172 (72.10%)	
Perceived community violence (mean, SD)	9.08 (2.03)	8.90 (1.86)	8.98 (1.98)	0.006
Receiving remittance (*N*, %)				
Yes	113 (5.43%)	65 (6.12%)	178 (5.69%)	0.398
No	1,951 (94.51%)	922 (93.62%)	2,873 (94.18%)	
Not reported	1 (0.05%)	2 (0.1%)	3 (0.15%)	
Food insecurity: HFIAS (mean, SD)	6.52 (2.07)	5.54 (2.02)	6.20 (2.10)	<0.001
Discrimination (mean, SD)	1.52 (4.77)	1.96 (4.86)	1.67 (4.81)	0.023

N is unweighted. Means and percentages are survey-weighted. p-values are from survey-adjusted t tests and χ^2^ tests.

^*^Sampled refugees who displaced to South Sudan.

Despite similar depressive symptom levels, substantial differences were observed in structural and contextual characteristics. Refugees were overwhelmingly camp-based (89.3%), whereas host community members primarily resided in regular settlements (86.5%). Citizenship patterns aligned with population type, with host community members largely South Sudanese (98.7%) and refugees predominantly Sudanese (80.0%) with smaller proportions from the Democratic Republic of Congo, Central African Republic, Ethiopia, and other countries. Refugees were substantially more likely to report as the recipient of social protection program (53.2%) compared with host community members (10.6%). Self-rated health differed in the expected direction, with refugees more often reporting better health categories, while material hardship was widespread in both groups: most respondents rated their household as below minimum, and coping based food insecurity levels were similarly elevated across populations. Safety related measures showed a more nuanced pattern, with refugees more likely to report feeling safe (74.1 vs. 68.7%) while also reporting slightly higher perceived community violence, underscoring how subjective safety can coexist with elevated ambient insecurity.

### 3.2 Predictive modeling of depressive symptoms

To evaluate the joint predictive contribution of health, socioeconomic, safety, and contextual factors to depressive symptoms, we compared the out-of-sample performance of three modeling approaches: gradient-boosted trees (XGBoost), Random Forest, and a linear Elastic Net baseline. Model performance was assessed using 10 × 5 nested cross-validation to minimize overfitting and ensure robust generalization. Under this framework, the gradient-boosted tree model demonstrated the strongest out-of-sample predictive performance among the models evaluated (Table 2A). XGBoost achieved an average RMSE of 4.47 (SD 0.25) and MAE of 3.39 (SD 0.20), explaining 24.7% (SD 3.8%) of variance in PHQ scores across outer folds. Random Forest showed closely comparable performance, with an RMSE of 4.51 (SD 0.23), MAE of 3.46 (SD 0.18), and *R*^2^ of 0.237 (SD 0.040). In contrast, the Elastic Net baseline performed substantially worse, with higher prediction error (RMSE 4.77, SD 0.27; MAE 3.69, SD 0.20) and lower explained variance (*R*^2^ 0.144, SD 0.034).

**Table 2A:** Model performance under 10 × 5 nested cross-validation (mean ± SD).

Model	MAE	RMSE	*R* ^2^
XGBoost	3.387 ± 0.197	4.473 ± 0.247	0.247 ± 0.038
Random Forest	3.455 ± 0.184	4.506 ± 0.232	0.237 ± 0.040
Elastic Net	3.691 ± 0.196	4.770 ± 0.270	0.144 ± 0.034

Panel A shows mean ± SD across outer folds; lower RMSE/MAE and higher R^2^ indicate better performance. Panel B compares fold-wise RMSE using paired Wilcoxon signed-rank tests; Δ = comparator – XGBoost.

Pairwise comparisons of outer-fold RMSE using Wilcoxon signed-rank tests further quantified these differences (Table 2B). Compared with Elastic Net, XGBoost demonstrated a statistically significant reduction in prediction error (*V* = 55, *p* = 0.006), corresponding to a median RMSE reduction of 0.320 PHQ points (IQR 0.180), or a 6.96% median reduction (IQR 3.48%). In comparison with Random Forest, XGBoost also showed a modest but statistically significant performance advantage (*V* = 51, *p* = 0.019), with a median RMSE reduction of 0.031 PHQ points (IQR 0.056), equivalent to a 0.71% median reduction (IQR 1.30%).

**Table 2B:** Paired Wilcoxon signed-rank tests on outer-fold RMSE.

Comparison	*V*	*p*-Value	Median ΔRMSE	IQR ΔRMSE	Median %Δ	IQR %Δ
XGBoost vs. Elastic Net	55	0.006	0.320	0.180	6.960	3.480
XGBoost vs. Random Forest	51	0.019	0.031	0.056	0.711	1.300

Δ = comparator – XGBoost; positive Δ means XGBoost has lower error. Tests are based on paired outer-fold RMSE values (n = 10 folds).

Based on these results, XGBoost was selected as the final model for interpretability analyses.

### 3.3 Relative importance and heterogenous effects of predictors

SHAP analysis of the final XGBoost model identified health status as the dominant predictor of depressive symptoms (Table 3). Health status had a mean absolute SHAP value of 1.069, accounting for 19.3% of total model contribution overall, with a slightly stronger relative contribution among refugees (22.4%) than hosts (18.2%). Perceived community violence formed the second most influential predictor (mean |SHAP| 0.612; 11.1% overall), with greater relative importance among hosts (15.4%) than refugees (10.4%). Poverty level (0.531; 9.6%), age (0.517; 9.3%), and discrimination (0.508; 9.2%) comprised a subsequent tier of predictors with comparable contributions across groups. Food insecurity (0.478; 8.6%) and citizenship status (0.452; 8.2%) also demonstrated moderate influence, although citizenship was only applicable in the pooled model. Sense of safety (0.417; 7.5%), rurality (0.368; 6.7%), and sex (0.359; 6.5%) contributed modest but consistent shares of total importance. Received social protection (0.187; 3.4%) and remittances (0.036; 0.7%) had comparatively limited influence. Overall, predictor rankings were broadly stable across refugee and host models, with variation primarily in the relative magnitude of contributions rather than in the ordering of dominant factors.

**Table 3:** Global feature importance for depressive symptom prediction based on SHAP values, overall and by population group.

Predictor	Mean |SHAP| (Total)	Relative importance %		
		Total, %	Host, %	Refugee, %
Health status	1.069	19.323	18.236	22.432
Perceived community violence	0.612	11.051	15.351	10.386
Poverty level	0.531	9.597	9.071	11.135
Age	0.517	9.339	10.876	9.817
Discrimination	0.508	9.175	10.604	9.686
Food insecurity	0.478	8.645	11.962	8.147
Citizenship	0.452	8.159	—	—
Sense of safety	0.417	7.537	5.844	9.380
Rurality	0.368	6.652	6.479	7.622
Sex	0.359	6.480	7.612	6.779
Received social protection	0.187	3.387	3.113	3.974
Receiving remittance	0.036	0.654	0.852	0.643

To assess potential heterogeneity by population type, we compared SHAP based relative importance profiles between refugees and host community members. Self-rated health remained the leading predictor in both groups (Host 18.2% vs. Refugee 22.4%). Several exposures differed in magnitude, with perceived community violence and food insecurity contributing more strongly among hosts (15.4 and 12.0%) than refugees (10.4 and 8.1%), whereas sense of safety contributed more strongly among refugees (9.4%) than hosts (5.8%). Overall, the dominant predictors in both groups reflected shared material and protection related gradients rather than displacement status itself, supporting pooled modeling to identify common structural correlates of depressive symptom severity.

**Figure 2:**
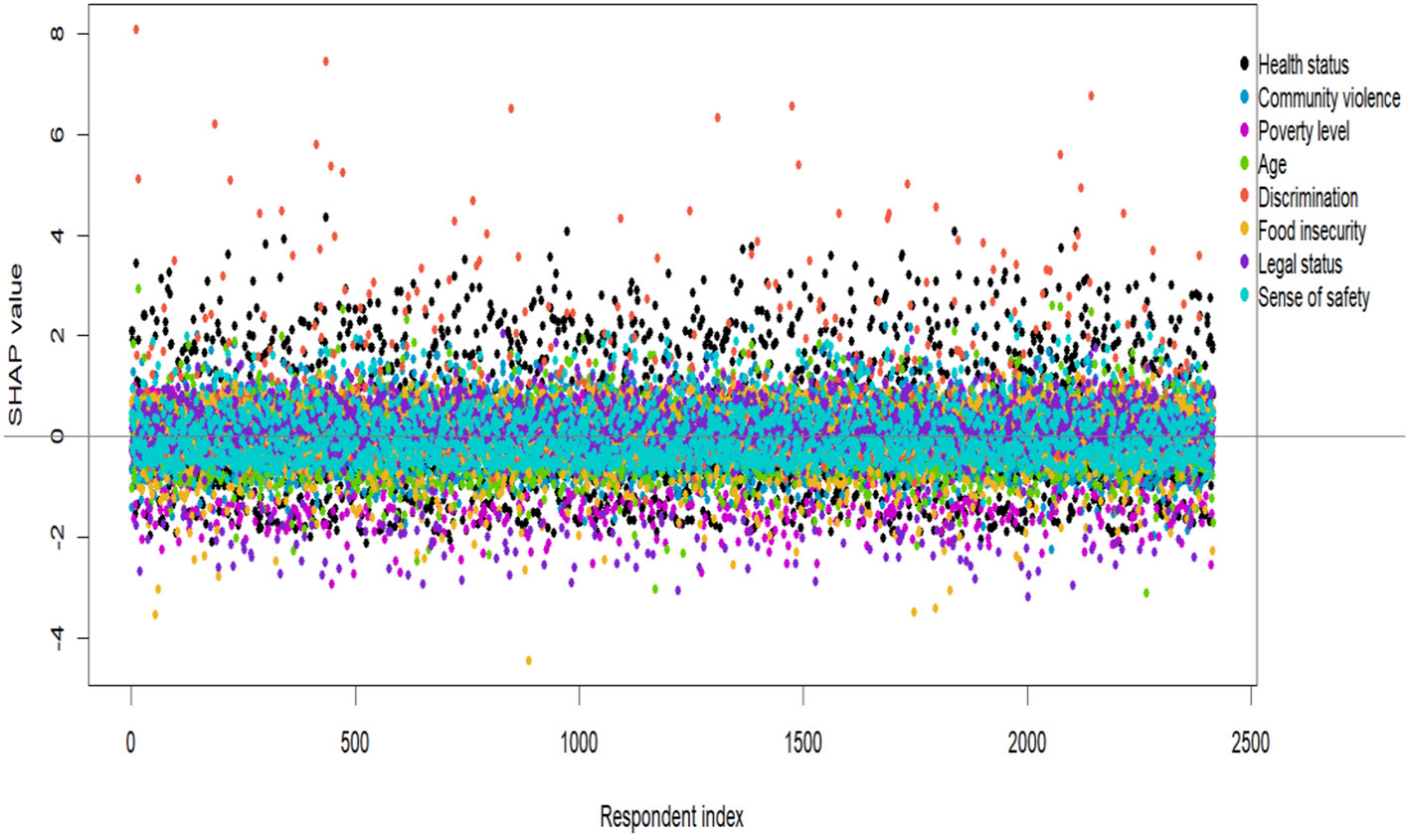
SHAP contributions of the top predictors in the XGBoost model for PHQ-9. Each point represents a participant-specific SHAP value (Δ predicted PHQ-9) for a given predictor. The horizontal line at zero denotes no contribution. Predictors are ranked by global importance, with health status, poverty level, sense of safety, and community violence showing the largest and most variable effects. Wider vertical spreads indicate stronger and more heterogeneous influences across individuals.

**Figure 3:**
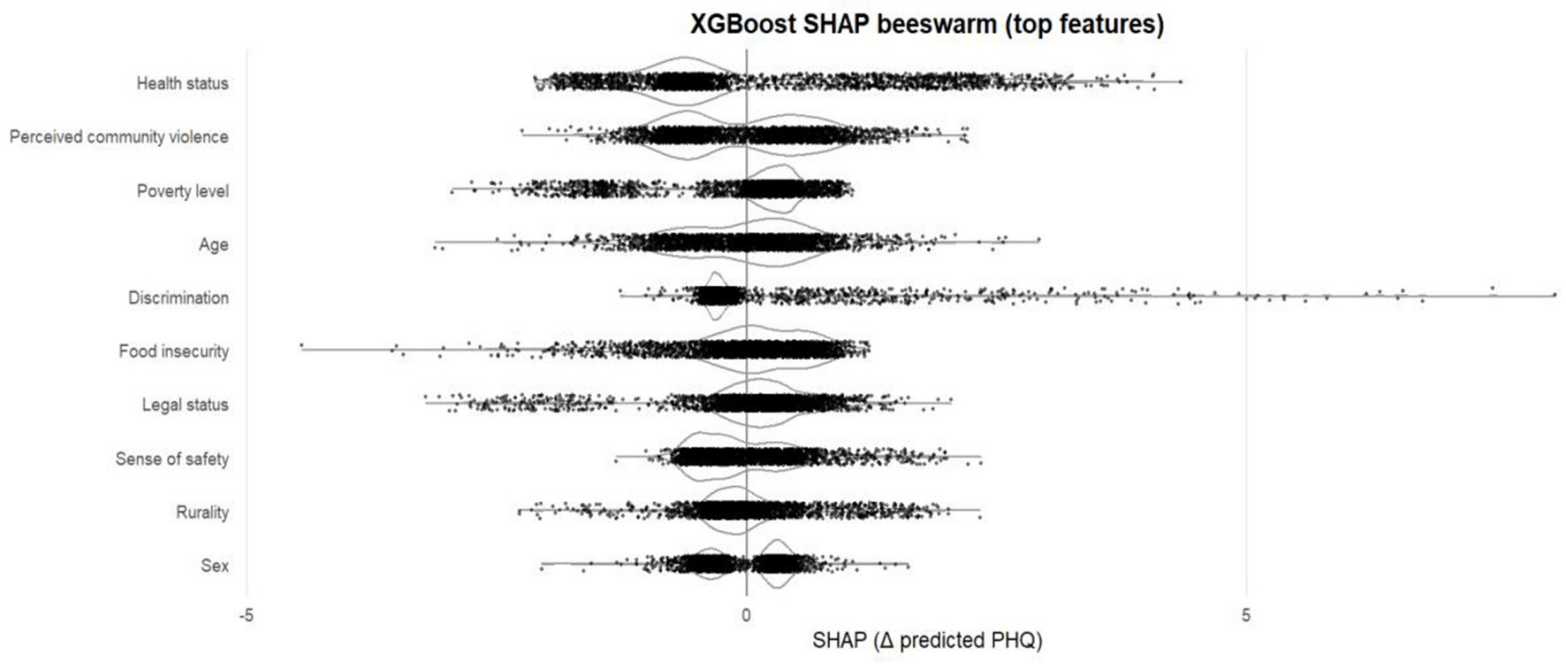
SHAP beeswarm plot of top predictors in the XGBoost model. Each point shows a participant-specific SHAP value (Δ predicted PHQ). Features are ordered by global importance.

Figure 2 displays the distribution of SHAP values from the final XGBoost model across all participants and all predictors. The x axis indexes participants. For each participant there are multiple points, one per predictor, showing how much that predictor moved the person's predicted PHQ up or down relative to the model baseline. Values above zero increased the prediction and values below zero decreased it. The dense band around zero means most predictor contributions are small for most people. The positive and negative tails up to roughly three PHQ points show that some predictors have large effects for a subset of participants. Colors distinguish predictors but this figure is not meant to identify which predictor is which (use Figure 3 for feature ranking and direction).

Figure 3 presents the SHAP beeswarm plot from the final XGBoost model predicting PHQ-9 depressive symptom severity. Each dot represents one participant, with the x-axis showing that feature's SHAP value, or contribution to the model's predicted PHQ score. Features are ordered by their mean absolute SHAP value, meaning those at the top have the greatest overall influence on predictions. The plot indicates that depressive symptom severity is most strongly structured by health and protection-related stressors. Self-rated health shows the largest magnitude and widest dispersion of SHAP values, suggesting a pronounced gradient in predicted symptoms across levels of perceived health and substantial heterogeneity in how health status relates to depressive severity. Perceived community violence and poverty level also display broad SHAP distributions that are largely shifted toward positive values, consistent with higher insecurity and economic strain contributing to higher predicted PHQ-9 scores. Discrimination exhibits a marked right-tail, indicating that while many participants show small or near-zero contributions, a subset experiences disproportionately large increases in predicted symptoms, consistent with concentrated psychosocial harm among those most exposed. In contrast, legal status and rurality contribute more modestly and with narrower SHAP ranges, implying that contextual positioning matters, but less than the shared material and protection environment in differentiating depressive symptom severity.

### 3.4 Sensitivity analyses addressing measurement overlap

Sensitivity analyses were conducted to assess potential overlap between self-rated health and somatic components of the PHQ-9. In models predicting the full PHQ-9 score, inclusion of self-rated health substantially improved predictive performance (*R*^2^ = 0.446 vs. 0.349; RMSE 3.83 vs. 4.15), corresponding to a 7.73% reduction in RMSE and a Δ*R*^2^ of 0.097. Aggregated SHAP values indicated that self-rated health accounted for 11.6% of total feature importance under this specification. To evaluate whether this contribution was driven primarily by overlapping somatic symptom content, we constructed a PHQ-7 affective-cognitive score by removing the sleep and fatigue items and re-estimated the identical modeling pipeline. Self-rated health remained a strong predictor (*R*^2^ = 0.417 vs. 0.338; RMSE 2.99 vs. 3.18; Δ*R*^2^ = 0.079), corresponding to a 6.15% reduction in RMSE. Notably, the relative SHAP importance of health increased to 15.4% under the PHQ-7 specification, indicating that its predictive contribution was not attenuated after removal of somatic items. For the two-item somatic subscore, inclusion of self-rated health yielded a smaller incremental improvement (*R*^2^ = 0.316 vs. 0.272; RMSE 1.28 vs. 1.32; Δ*R*^2^ = 0.045; 3.11% RMSE reduction). Health accounted for 11.5% of SHAP-based importance in this restricted model, consistent with partial measurement overlap between perceived general health and physical symptom reporting.

## 4 Discussion

We examined predictors of depressive symptoms among refugees and host community members in South Sudan using explainable machine learning. Because depressive symptom severity did not differ meaningfully by population type, analyses were conducted using a single model, with predictors interpreted as operating within a shared structural environment shaped by prolonged displacement, economic contraction, and recurrent insecurity. Sensitivity analyses confirmed that predictor rankings and directional effects were stable across refugee and host subsamples, indicating that legal status did not fundamentally alter the structural patterning of depressive symptoms. These conditions reflect the broader socioeconomic and political context described in the SDoH framework, including fragile governance, disrupted labor markets, weak social protection, and macroeconomic instability that structure access to livelihoods, services, and mobility. In this setting, socioeconomic position is produced through institutional and policy arrangements rather than legal labels alone, locating individuals within constrained opportunity structures that shape health risks and resources. This also aligns with evidence from conflict-affected settings showing that mental health burden is driven less by categorical status than by cumulative hardship and constraints on daily functioning and future prospects (36, 38, 56). This pattern emphasize that structural and material gradients often outweigh legal or migratory classifications in shaping population-level mental health outcomes (40). Population-level estimates similarly indicate persistent prevalence of common mental disorders across conflict settings, with variation driven primarily by exposure and living conditions rather than labels alone (22). Using explainable machine learning allowed us to move beyond average associations and identify which structural conditions most strongly differentiated depressive symptom burden within this high-adversity context (42, 57).

Within this shared structural context, self-rated health status emerged as the dominant predictor of depressive symptoms in the SHAP analysis. In South Sudan's current humanitarian context, this pattern reflects more than comorbidity. Sensitivity analyses excluding the two somatic PHQ items yielded substantively similar findings, with self-rated health retaining substantial predictive contribution. This suggests that while somatic overlap is plausible, the predictive dominance of self-rated health cannot be explained solely by shared symptom content. Rather, health status appears to function as a marker of embodied precarity, integrating chronic illness burden, functional limitation, and constrained access to care within a fragile health system (56). In turn, mental distress is closely tied to embodied vulnerability and functional limitation, particularly where illness reduces livelihood participation, access to care is disrupted by insecurity and weak health systems, and households absorb the economic consequences of poor health. The prominence of health status can be further interpreted through the lens of differential vulnerability and differential consequences. In high adversity contexts, risks cluster and interact, increasing susceptibility to the psychological effects of physical illness. At the same time, the social and economic consequences of ill health are amplified in settings with limited social protection and fragile labor markets, where even minor functional decline may precipitate livelihood loss. These feedback processes intensify depressive symptom severity and help explain the stratifying role of health status in our models. Consistent with this interpretation, prior studies in South Sudan document substantial depressive symptom burden alongside material deprivation and cumulative trauma exposure, with mental health closely linked to ongoing adversity (16, 18). The heterogeneity observed in SHAP contributions indicates that health status stratifies risk unevenly, acting as a decisive driver of symptoms for some individuals while playing a more modest role for others. This pattern supports a long-standing but often under-implemented implication for global mental health practice: in fragile settings, mental health gains are likely to depend on routine integration into primary care and chronic illness management, rather than reliance on standalone psychological programming alone (58, 59).

Beyond individual health, a second cluster of predictors, such as poverty level, sense of safety, and perceived community violence, contributed at similar magnitudes, underscoring depression as a condition embedded in structural stress rather than an isolated psychological response. This configuration closely reflects the daily stressors framework, which emphasizes that ongoing deprivation and insecurity frequently mediate, compound, or even outweigh the mental health effects of discrete traumatic events (36). Interpreted alongside the descriptive characteristics of the sample, this pattern suggests that material hardship is not a peripheral covariate but a central organizing condition of distress in both displaced and non-displaced communities. The juxtaposition of higher reported safety alongside slightly higher perceived community violence among refugees is particularly informative, as it points to layered meanings of security. Subjective safety may reflect immediate predictability and protection within one's local environment, including camp governance and proximity to services, while perceived violence may capture ambient threat within the broader social and political ecology. This distinction has direct implications for intervention design, suggesting that improvements in local protection may not translate into reduced perceptions of violence without broader conflict mitigation and community safety efforts.

Along with these structural stressors, the contributions of legal status and rurality point to the role of opportunity structures and access pathways in shaping depressive symptoms. Legal status conditions entitlements, mobility, and access to assistance, while rurality often reflects infrastructural marginalization, distance to care, and limited market integration. These patterns are consistent with prior research in South Sudan linking socioeconomic conditions and war-related exposures to depression and comorbidity (16, 17). At the same time, the relatively modest contribution of social protection across both refugee and host models, despite substantial differences in exposure observed descriptively, is a policy-relevant signal. Existing assistance may be insufficient in adequacy, regularity, or scope to buffer depressive symptoms against dominant drivers such as poor health and insecurity. Evidence from humanitarian and low-income settings suggests that cash and related transfers can improve material wellbeing, but mental health effects are uneven and depend on program design, reliability, and links to health and protection services (60, 61). In South Sudan's context of macroeconomic volatility, inflation and market disruption may further erode the buffering potential of transfers.

While the model identified several dominant predictors, food insecurity and emerged as mid-tier contributors rather than leading predictors in the primary PHQ-9 specification. Both are well-established predictors of depressive symptoms across humanitarian, low-income, and displaced populations, with evidence linking chronic food deprivation and social exclusion to elevated depression and psychological distress (37, 38). In this sample, food insecurity was widespread and highly concentrated, which may have limited its ability to distinguish sharply between individuals at different levels of depressive symptom severity within an already high-adversity environment. Although discrimination demonstrated a measurable contribution to prediction (i.e., 9.2% of total SHAP importance), its distribution suggests that effects may be concentrated among a subset of highly exposed individuals rather than operating uniformly across the population. Low overall endorsement may reflect floor effects, normalization of social harm, or limited sensitivity of available items rather than the absence of stigmatizing experiences. Qualitative and measurement work in South Sudan highlights locally salient idioms of distress, including “overthinking,” and context-grounded expressions of social harm that may not be fully captured by standard instruments (19, 21). Overall, these patterns reflect distributional and measurement constraints rather than substantive irrelevance and underscore the need for future research and monitoring tools that can capture meaningful gradients of deprivation and social harm in settings where extreme exposure is common.

The findings have direct implications for policy and programming in the conflict- and climate-stressed environment described in the Introduction. First, because symptom burden was similar across population type while predictors mapped strongly onto shared structural conditions, area-based responses serving both refugees and host communities are likely to be more efficient and equitable than approaches that assume fundamentally different mental health etiologies by legal status. This aligns with long-standing guidance to situate Mental Health and Psychosocial Support (MHPSS) within layered, multi-level systems of care (62). In humanitarian settings, MHPSS encompasses interventions ranging from community-based and family-focused supports to focused psychosocial care and clinical services, and it is most effective when coordinated with protection, primary health care, and livelihood programming that shapes the social and material conditions of distress (37, 59). Second, the prominence of health status points to integrated care as a high-yield entry point. Screening and stepped care embedded within primary care, maternal health, and chronic disease platforms may be particularly effective in identifying and reaching those at greatest risk. Third, the salience of poverty, safety, and violence supports combined intervention packages, including predictable social protection with adequate purchasing power, protection interventions that reduce daily threat and strengthen perceived safety, and community-level strategies that address violence and support social cohesion. Fourth, SHAP-based heterogeneity supports more precise targeting without reliance on stigmatizing categories. Programs can identify individuals whose risk profiles show large predicted contributions from modifiable domains, such as barriers to health care access or safety concerns, and allocate more intensive support accordingly. This approach is consistent with interpretability guidance emphasizing transparent translation of complex models into actionable program levers (41, 43) and with reviews noting that machine learning in mental health is most useful when it advances practical decision support rather than classification alone (42).

At the same time, several limitations should be considered when interpreting these findings, most of which reflect structural and measurement constraints typical of conflict- and displacement-affected settings rather than shortcomings unique to this study. First, the cross-sectional design limits causal inference, particularly in disentangling the directionality between health status, material hardship, and depressive symptoms. However, the objective of this analysis was not causal attribution but identification of dominant and differentiating risk patterns within a high-adversity context, for which the applied approach is well-suited. Second, PHQ scores capture depressive symptom severity rather than clinical diagnoses, and some overlap between somatic symptoms and self-rated health is unavoidable in settings where physical illness, fatigue, and functional impairment are widespread. Rather than indicating measurement bias alone, the prominence of health status likely reflects the lived reality of distress in South Sudan, where mental and physical suffering are deeply intertwined and access to treatment for chronic conditions is limited. This convergence underscores the relevance of integrated care models rather than undermining the substantive interpretation of findings. Third, several salient stressors, including trauma exposure, bereavement, recent displacement, and climate-related shocks, were not incorporated into the final model. Some domains were measured only in limited form or exhibited low endorsement in this survey wave, while others lacked temporal or contextual specificity to capture cumulative and dynamic risk processes, reducing their suitability for stable predictive modeling Finally, although explainable machine learning improves transparency relative to black-box approaches, SHAP values remain conditional on the variables measured and the structure of the model. Interpretability does not eliminate the need for theory-driven measurement or contextual knowledge. Instead, it should be viewed as complementary, offering a systematic way to surface dominant structural drivers and heterogeneity that can inform hypothesis generation, program targeting, and future longitudinal research.

Despite these limitations, this study provides a clear picture of how depressive symptoms are structured within a context of prolonged conflict, displacement, and economic instability. Poor health, material hardship, insecurity, and violence emerged as shared and dominant drivers of distress across refugees and host community members, underscoring the limits of status-based approaches to mental health intervention. By pairing predictive modeling with transparent interpretation, this analysis demonstrates how explainable machine learning can inform integrated, area-based responses that link mental health support with primary care, social protection, and protection systems. In settings of pervasive adversity, such approaches offer a practical path toward translating complex evidence into actionable strategies for mental health policy and programming.

## Data Availability

The data analyzed in this study is subject to the following licenses/restrictions: this study uses de-identified individual- and household-level data from Forced Displacement Survey (FDS)—South Sudan 2023. The dataset was collected and is managed by the United Nations High Commissioner for Refugees (UNHCR) and was made available to the authors under a formal data use agreement. The data are not publicly available, but access may be requested directly from UNHCR in accordance with their data-sharing procedures. Requests to access these datasets should be directed to https://microdata.unhcr.org/.
