# Supplementary material for: Structural determinants of depressive symptoms among refugees and host communities in South Sudan: evidence from explainable machine learning
Source: Front Public Health. 2026 Apr 14;14:1778986. doi: 10.3389/fpubh.2026.1778986 (PMC13120975; doi:10.3389/fpubh.2026.1778986)
Supplement: Supplementary file 1 [file Table_1.DOCX]

# Supplementary Material

**Table 1S. Missing Data Table**

| Variable | Missing (N) | % Missing |
| --- | --- | --- |
| PHQ | 0 | 0.0% |
| Age | 67 | 2.19% |
| Health status | 1 | 0.03% |
| Biological sex | 1 | 0.03% |
| Marital status | 14 | 0.46% |
| Rurality | 2 | 0.07% |
| Residence | 4 | 0.13% |
| Citizenship | 77 | 2.52% |
| Received social protection | 9 | 0.29% |
| Poverty Level | 175 | 5.73% |
| Sense of safety | 116 | 3.80% |
| Perceived community violence | 171 | 5.60% |
| Remittance (Receiving remittance) | 1 | 0.03% |
| Discrimination | 200 | 6.5% |
| Food Insecurity (HFIAS) | 13 | 0.4% |
